# From Sensing to Sense-Making: A Framework for On-Person Intelligence with Wearable Biosensors and Edge LLMs

**DOI:** 10.3390/s26072034

**Published:** 2026-03-25

**Authors:** Tad T. Brunyé, Mitchell V. Petrimoulx, Julie A. Cantelon

**Affiliations:** 1 Center for Applied Brain and Cognitive Sciences, Tufts University, Medford, MA 02155, USA; 2 U.S. Army DEVCOM Soldier Center, Natick, MA 01760, USA

**Keywords:** wearable biosensors, artificial intelligence, edge computing, large language models, multimodal sensing, decision support, human–machine integration

## Abstract

**Highlights:**

**What are the main findings?**

- A key bottleneck in real-world wearable sensing is the transformation of noisy physiological signals into actionable decisions; a cognitive co-pilot architecture is proposed linking sensing, probabilistic state estimation, LLM-based contextual reasoning, and attention-aware intervention.
- Local, uncertainty-aware, edge-deployed reasoning is a necessary architectural condition for trustworthy decision support in high-stakes environments, and several research gaps exist to achieve this goal.

**What are the implications of the main findings?**

- Effective wearable-AI systems will require integrated sociotechnical design combining sensor validation, uncertainty-calibrated inference, grounded LLM reasoning, and cognitive-engineering-driven interface policies.
- Beyond model accuracy, progress should be evaluated in downstream human outcomes (trust calibration, workload, decision quality, long-term reliance), reframing success as improved human–system integration.

**Abstract:**

Wearable biosensors increasingly stream multi-channel physiological and behavioral data outside the laboratory, yet most deployments still end in dashboards or threshold alarms that leave interpretation open to the user. In high-stakes domains, such as military, emergency response, aviation, industry, and elite sport, the constraint is rarely data availability but the cognitive effort required to convert noisy signals into timely, actionable decisions. We argue for on-person cognitive co-pilots: systems that integrate multimodal sensing, compute probabilistic state estimates on devices, synthesize those states with task and environmental context using locally hosted large language models (LLMs), and deliver recommendations through attention-appropriate cues that preserve autonomy. Enabling conditions include mature wearable sensing, edge artificial intelligence (AI) accelerators, tiny machine learning (TinyML) pipelines, privacy-preserving learning, and open-weight LLMs capable of local deployment with retrieval and guardrails. However, critical research gaps remain across layers: sensor validity under real-world conditions, uncertainty calibration and fusion under distribution shift, verification of LLM-mediated reasoning, interaction design that avoids alarm fatigue and automation bias, and governance models that protect privacy and consent in constrained settings. We propose a layered technical framework and research agenda grounded in cognitive engineering and human–automation interaction. Our core claim is that local, uncertainty-aware reasoning is an architectural prerequisite for trustworthy, low-latency augmentation in isolated, confined, and extreme environments.

## 1. Introduction

Over the last fifteen years, personal biosensing has progressed from sporadic measurement to continuous high-volume digital exhaust [1,2,3]. Early work in mobile and wearable sensing established the procedures for continuous collection, opportunistic sampling, on-device preprocessing, and context inference, which now underpin commercial wearables and occupational monitoring programs [4]. Yet a persistent mismatch remains between what our systems can measure and what users and decision-makers may need in the moment [5,6]. Heart rate variability (HRV), photoplethysmography (PPG), electrodermal activity (EDA), accelerometry, skin temperature, respiration, sleep staging, and (in some contexts) EEG and eye tracking are all increasingly feasible at the edge. But high-stakes failures rarely occur because decision-makers lacked access to raw time series data; failures more likely occur because decision-makers were forced to interpret ambiguous signals under time pressure, distraction, uncertainty, and competing operational goals.

In a typical biosensing deployment, sensors stream to a hub, features are computed, classifiers label discrete states, and outputs are visualized as readiness scores, traffic lights, body batteries, gauges, flags, or other alerts. The final step, translating from *state* to *action*, is often left under-specified [7,8]. Even when a model correctly classifies and alerts to high stress or fatigue, the user still faces a gap that limits actionability [9]: How does this state interact with the present phase of a task or goal? Is it transient or trending? Is it risky now, or merely informative? What intervention is feasible given operational or contextual constraints? What is the cost of intervening incorrectly?

Cognitive engineering has long emphasized that the human is not a peripheral user, but a coupled component in a sociotechnical system [10]. Classic constructs such as limited working memory, attentional bottlenecks, situational awareness, and mental workload help determine whether information is noticed, trusted, integrated, and acted upon [5,9,11,12]. These theories converge on a practical implication: high-stakes interventions must be considered a decision-support partner that manages attention, uncertainty, and timing. We argue that this is where locally hosted LLMs become potentially useful. Namely, as computational mechanisms for contextual synthesis, explanation, and dialog, provided they are constrained, grounded, and audited [13,14,15]. Our paper is motivated by recent advances in edge artificial intelligence (AI) and local language-model deployment, including runtime-efficient inference for edge LLMs, improved post-training quantization and compression methods, and a growing literature on human-AI collaboration and wearable sensing pipelines [7,15,16,17,18].

**Figure 1:**
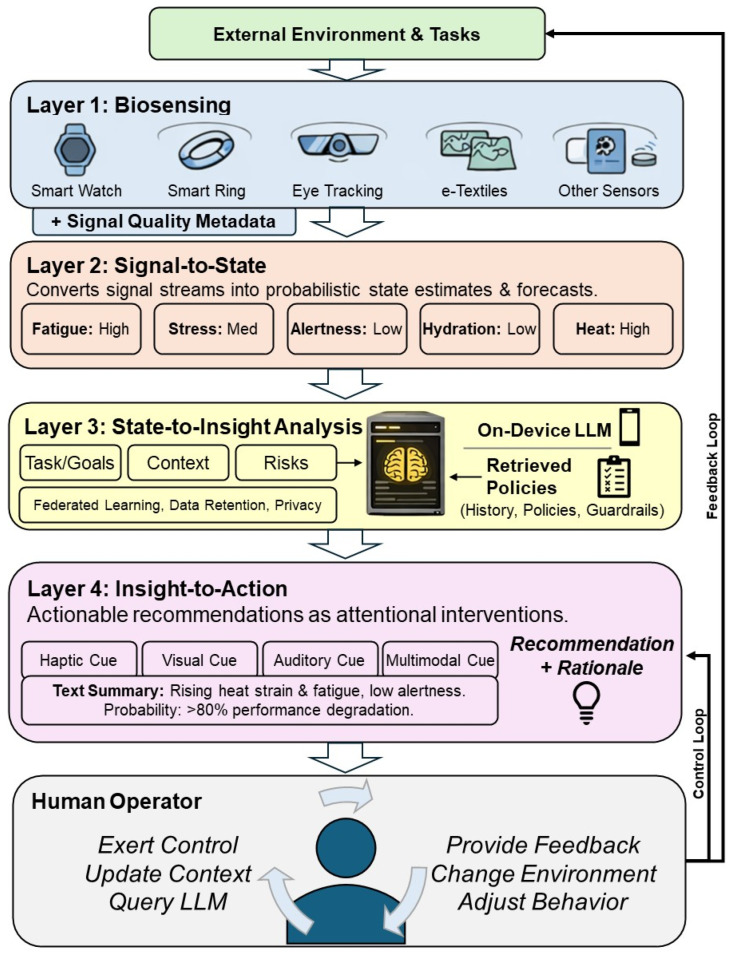
Conceptual framework for an on-person cognitive co-pilot. Multimodal signals from the external environment and task context are acquired through a biosensing layer (wearables, e-textiles, eye tracking, and other sensors), processed on-device into probabilistic state estimates (signal-to-state), synthesized with task and historical context to generate actionable insights (state-to-insight), and delivered through modality-appropriate cues (insight-to-action). A continuous feedback and control loop supports context injection, natural-language querying, user override, and system calibration to promote appropriate reliance.

In this opinion paper, we do not present a new algorithm or empirical system evaluation; rather, we synthesize existing technical and cognitive science studies into a conceptual framework and research agenda. We organize this agenda around a four-layer framework: (1) multimodal sensing, (2) probabilistic state estimation, (3) contextual reasoning, and (4) attentional action, clarifying where technical and human-factor risks emerge (Figure 1).

The novelty of this Perspective article is not the introduction of a new sensor, classifier, or standalone LLM application. Rather, it is the explicit integration of four elements that are often treated separately in the literature: (i) multimodal, quality-aware biosensing, (ii) uncertainty-calibrated probabilistic state estimation, (iii) grounded local reasoning that translates state estimates into context-sensitive options and explanations, and (iv) attention-aware interventions designed to preserve human autonomy and calibrated trust. Existing work has often focused on one of these components in isolation, such as wearable sensing, edge inference, dashboarding, threshold alarms, or LLM-enabled explanation. Our contribution is to position these as parts of a single sociotechnical architecture for on-person cognitive co-pilots and identify the technical and human-factor dependencies that must be solved jointly for trusted deployment in high-stakes environments.

## 2. Edge Computing as an Enabler

Edge computing is often motivated by latency and bandwidth optimization. In high-stakes biosensing, it may be more accurately framed as both necessary (i.e., given network availability and/or security constraints) and trust enabling. Satyanarayanan argued that edge computing emphasizes proximity to the data source, reduced latency, and improved robustness when connectivity is limited, properties that map directly onto isolated, confined, and extreme (ICE) environments [19]. Shi and colleagues likewise highlighted the technical imperatives: real-time processing near sensors, resilience to network disruption, and the need to manage heterogeneity and mobility [20].

These imperatives matter because many occupational contexts either cannot tolerate a cloud dependency (submarines, mines, disaster zones, contested environments) or cannot accept the privacy and governance implications of streaming raw biometrics off-person [21,22,23]. Local inference changes the model: sensitive data can be reduced to features or probabilistic state estimates, stored briefly, and protected via device security primitives. It also changes the interaction model: if inference must wait on a network round trip, the system will either be ignored (too slow) or will push users back to conservative alarms (too brittle).

In parallel, hardware and software ecosystems have matured to make on-device inference practical. Tiny machine learning (TinyML) and embedded inference pipelines have demonstrated that compressed neural networks can run within tight power envelopes when the full pipeline (i.e., sampling, preprocessing, model, postprocessing) is integrated [24]. Open-weight LLMs now provide a controllable substrate for on-device reasoning, especially when combined with retrieval, tool use, and strict output schemas [25,26]. Below, we describe key layers of a system that transforms sensing into sense-making.

### 2.1. Layer 1: Multimodal Sensing

The sensing layer is critical, but motion, sweat, temperature extremes, poor contact, and electromagnetic noise can all degrade signal quality and induce systematic bias [27,28]. Photoplethysmography (PPG) is a canonical example: while it is attractive for heart rate (HR) and peripheral capillary oxygen saturation (SpO_2_) estimation, motion artifacts and contact changes can dominate the waveform during physical activity, making accuracy highly context dependent [29,30]. Similar challenges exist for electrodermal activity (EDA; sweat and temperature sensitivity) [31], skin temperature (environmental coupling) [32], and electroencephalography (EEG; electrode stability and artifact contamination) [33].

This is why the most credible architectures embrace redundancy and fusion. Human activity recognition and context inference research has repeatedly shown that multi-sensor combinations, such as IMU + location + physiological channels, outperform any single stream and provide robustness under partial failure [34,35,36,37]. The same logic applies to readiness and risk monitoring: a plausible “fatigue” estimate might integrate sleep history, HRV dynamics, movement micro-patterns, and behavioral markers (e.g., response variability); it would not treat any one feature as decisive [38].

Wearable form factors are expanding the design space. E-textiles and smart garments promise distributed sensing that may be more comfortable and less obtrusive than point devices, while also enabling multi-site measurement (e.g., respiration via chest expansion, EMG at muscle groups). Reviews have detailed both the opportunity and the practical limitations: washability, durability, signal stability, and integration with power and data pathways [39,40,41]. Likewise, noninvasive biochemical sensing, particularly with sweat-based analytes, has advanced rapidly, with work surveying electrochemical and microfluidic approaches for electrolytes, metabolites, and stress-relevant markers [42]. The near-term point is not that a single biomarker (e.g., cortisol) will be perfectly measured in the field tomorrow; rather, that the sensing layer is increasingly capable of providing multi-modal proxies for state inference.

A key technical implication is that Layer 1 should not merely collect data from sensors. It should produce metadata outputs related to signal quality, such as contact confidence, motion intensity, ambient interference, and missingness and artifact likelihood [43,44]. Without explicit quality modeling, downstream classifiers and LLM reasoning will conflate physiology with artifact and degrade trust when the system fails in what might have been predictable, repeatable ways.

### 2.2. Layer 2: Probabilistic State Estimates

Layer 2 is where raw streams become interpretable state variables, and where many current systems stop. In occupational settings, it is not sufficient to label stress as high. The system must quantify uncertainty and detect drift. Activity recognition research has long wrestled with these issues, including non-independent and identically distributed (non-IID) data across individuals, context-dependent movement signatures, and sensor placement variability [35,45]. In real sensor deployments in occupational contexts, these are not edge cases—they are often the default.

Historically, signal-to-state pipelines relied on hand-engineered features (e.g., time-domain HRV metrics, frequency-domain power, EDA peaks, IMU statistics) paired with classical models [46,47,48]. Those methods remain valuable on-device because they are interpretable and computationally efficient; however, deep learning models (e.g., temporal convolutional neural networks/CNNs, long short-term memory/LSTMs, gated recurrent units/GRUs, and increasingly lightweight transformers) have demonstrated superior performance when trained on large, diverse datasets and when they can learn invariant features across contexts. The challenge is that field physiology is heterogeneous, and accuracy on a benchmark may not reflect real-world performance. Three technical priorities deserve emphasis in an expert-facing architecture:

- Fusion as Estimation. Feature concatenation can fail silently when one channel is corrupted. Fusion should be treated as a state estimation problem: combine multiple noisy observations into a posterior belief over latent states. Classical filters (e.g., Kalman variants, particle filters) and modern neural Bayesian approximations can serve this role; the critical design point is to produce a distribution (or at least calibrated confidence) in addition to a discrete label [49,50].
- Calibration and Abstention. A classifier that always outputs a label is dangerous in high-stakes contexts. Layer 2 should support abstention (i.e., insufficient confidence) and graceful degradation (i.e., automated fallback to simpler models when signals are poor) [51,52]. Providing users with uncertainty information can support calibrated trust in a system and organization.
- Continual Improvement. Federated learning provides a principled mechanism to update models across multiple devices while keeping training data local, aggregating parameter updates [53,54]. In practice, federated pipelines must still handle adversarial updates, heterogeneity, and auditability; but as an architectural concept, they align naturally with occupational privacy constraints.

Personalization is necessary because physiological baselines, behavioral signatures, and task responses vary substantially across users; of course, personalization must be bounded to avoid overfitting and silent model drift. In practice, safer personalization should rely on conservative mechanisms such as baseline normalization, threshold adjustment within predefined limits, calibration to recent within-user history, and periodic recalibration windows rather than unrestricted online updating of all model parameters. Drift detection should likewise be treated as a first-class function of Layer 2, using indicators such as persistent degradation in signal quality, shifts in feature distributions, instability in posterior estimates, or repeated disagreement between model expectations and observed data patterns [45,55]. When drift is detected, the system should not continue to produce recommendations in the same manner it would with confident inputs; instead, it should respond gracefully by lowering confidence, widening uncertainty bounds, increasing abstention frequency, falling back to a simpler or better-calibrated model when available, and, when necessary, requesting sensor adjustment, recalibration, or renewed baseline collection [51,52,56]. In high-stakes deployments, this type of bounded personalization and explicit drift response is preferable to maximizing short-term predictive fit at the cost of reduced transparency and trust.

By the end of Layer 2, the system should output a compact, semantically meaningful state vector with uncertainty. Concretely, the Layer 2 output should be something the system can treat as a best estimate of the user’s current state, along with how confident it is and whether conditions look outside what the model was trained for. For example, fatigue could be represented as an individualized fatigue estimate with a confidence range relative to baseline; heat strain as a risk probability with a confidence score and a drift flag; and abstention as an explicit “no recommendation” outcome when confidence is low and/or signal quality is poor. Uncertainty may reflect both measurement noise and model uncertainty under distribution shift, both of which are critical in field deployments. These state estimates may include hydration, fatigue, stress, heat strain, cognitive load, or motor stability, ideally with uncertainty estimates [57,58]. Here, *state* refers to a probabilistic latent variable inferred from multiple signals, not a direct measurement. Without uncertainty-aware state representations, downstream reasoning systems are forced to treat estimates as facts, amplifying error and overconfidence at higher layers. This state vector is the handoff point to Layer 3. Practically speaking, this handoff should be treated as a structured interface specification so that uncertainty, drift status, and abstention are preserved (and not collapsed into a single point estimate).

### 2.3. Layer 3: From States to Insights

The promise of Layer 3 is that an LLM can serve as a contextual synthesis engine, a mechanism for turning a state vector plus task/environment constraints into a structured recommendation and explanation, in natural language when needed, with a traceable data provenance [58,59,60]. Importantly, we do not propose that the LLM directly infers physiology or replaces the underlying state estimation. Layer 2 produces the core truth outputs (state estimates, uncertainty, and quality/drift indicators). Layer 3 is limited to structured synthesis, linking recommendations to trusted guidance, and dialog for clarification, while preserving uncertainty and supporting abstention when evidence is weak.

The role of the LLM is three-fold. First, for context integration, it binds the Layer 2 state vector to task phase, role constraints, environmental hazards, and historical patterns [57,61,62]. The result is not *fatigue is high*, but rather *fatigue is high relative to baseline, with rising trend and low recovery likelihood in the next 45 min*. For example, a fatigue estimate with high uncertainty during a high-stakes occupational exercise should yield a recommendation to monitor and re-assess (i.e., rather than to intervene immediately); an unconstrained language model might instead overconfidently narrate an urgent risk and prompt unnecessary disruption.

Second, for decision structuring, an LLM can translate latent estimates into action options (i.e., candidate courses of action), explicitly noting tradeoffs: intervene now (cost: time, mission impact), delay (cost: rising risk), or gather more information (cost: attention) [63,64]. A key design choice is to present options, not commands, unless immediate safety thresholds are crossed.

Third, explanations can support calibrated trust. Interpretability research is clear that an explanation must match the stakeholder’s needs, context, and timing [65,66,67,68]. In a high-stakes moment, a one-sentence rationale plus a confidence statement may be best; later, a detailed audit trail may be essential for learning (after-action review) and accountability.

These roles are only feasible for a local LLM if the system enforces evidence grounding, uses structured outputs, and treats the LLM as one component in a verifiable pipeline.

While promising, there are also inherent risks associated with LLM application. LLMs are prone to hallucination and overconfident narrative [69,70,71]. The technical response is to ground generation in retrieval and tools. Retrieval-Augmented Generation (RAG) is the canonical pattern: retrieve relevant, vetted documents (protocols, doctrine, medical guidelines, SOPs, user baselines) and constrain the model to synthesize from those sources [64,72,73,74]. In a cognitive co-pilot, RAG should be paired with strict output schemas (e.g., recommendation, rationale, confidence, data sources used, what would change my mind) and with refusal behaviors when evidence is insufficient.

Local deployment of LLMs is only feasible with compression. Open-weight model families (e.g., Llama, Gemma, Qwen) provide strong baselines for local reasoning and summarization, but real edge use typically requires quantization and efficient runtimes [25,75,76,77,78,79]. Tooling ecosystems such as llama.cpp and local runners make this practical on commodity devices, and quantization methods such as GPTQ and AWQ show how weights can be reduced with limited quality loss, though the impact on reliability under uncertainty remains an open research question for high-stakes applications [80,81]. For local reasoning modules, evaluation should include at minimum schema adherence, consistency across repeated identical queries, hallucination rate under ambiguous or conflicting retrieved inputs, abstention/refusal behavior when evidence is insufficient, and latency/energy cost per reasoning call.

### 2.4. Layer 4: From Insight to Action

Layer 3 is where insights are formed, and Layer 4 is where systems frequently succeed or fail in practice. Alarm fatigue in healthcare illustrates the hazard: even when alarms are technically correct, high frequency and low specificity can cause users to ignore them, silence them, or work around them [82,83]. In high-stakes work, frequent false positives erode trust and add workload; in contrast, false negatives create unjustified reassurance, and poorly timed prompts can disrupt attention during critical phases [84].

This is where cognitive engineering should dominate design decisions. Cognitive Load Theory predicts that interventions that add extraneous processing at the wrong time will degrade performance even when information is accurate [85]. Multiple Resource Theory predicts that modality choices matter: visual overlays can be costly during visually demanding tasks, whereas brief haptic signals or concise audio might be less interfering [86]. Situation Awareness theory suggests that displays should support projection and prioritization, not merely reporting [9,87].

Human–automation interaction research adds another set of hazards: automation bias (i.e., over-reliance on system outputs) and complacency (i.e., reduced monitoring when automation is present). Parasuraman and colleagues framed levels of automation as a spectrum that changes the human’s role from active controller to passive monitor; the risk is not simply too much automation, but poorly calibrated automation that shifts responsibility without maintaining engagement [5,88]. Empirical work has specifically linked decision aids to complacency and bias through attentional mechanisms [89,90]. That is, people look less, verify less, and accept more when automation appears authoritative [91]. Trust research further emphasizes that the goal is not maximal trust, but appropriate reliance, a dynamic alignment between system competence and user expectations [92,93].

Taken together, Layer 4 should be designed as a control policy for attention. It should regulate when the system speaks, how it speaks, and what it asks the human to do. Three design principles should be used. First, event gating reserves intrusive alerts for high-confidence, high-consequence conditions, and treats low-confidence states as monitoring or queryable. Second, modality matching maps message types to channels (e.g., haptic for urgent binary signals, audio for short directives, visual for reflective analysis). Finally, trust calibration couples recommendations with uncertainty, rationale, and (when feasible) counterfactuals (e.g., *I am concerned because X; I would relax this concern if Y improved.*).

To make this layer operational, the intervention policy can be framed as a mapping from system state and task context to an intervention decision. Relevant inputs include the posterior state estimate and associated uncertainty from Layer 2, signal-quality indicators, drift or out-of-distribution flags, task phase or operational tempo, workload proxies (e.g., movement intensity, task-event markers, or historical workload patterns), recent alert history, and modality availability. A simple baseline policy family can be defined in terms of gated intervention rules. For example, high-confidence and high-consequence conditions may trigger immediate alerts when interruption cost is low; moderate-confidence states may generate deferred or monitoring actions that surface information only at natural task boundaries; and low-confidence or drift-flagged conditions should increase abstention or request additional data rather than producing actionable recommendations. We return to this idea in Section 4.3. More sophisticated implementations could treat intervention timing as a utility optimization problem that balances expected benefit against interruption cost, or as a learnable policy (e.g., contextual bandit or reinforcement learning framework) that adapts cue timing over repeated deployments. Importantly, regardless of implementation method, the policy should preserve transparency by exposing the variables that influenced the intervention decision and by logging intervention outcomes for later audit and refinement [94].

### 2.5. A Formal Reference Architecture

To make the proposed framework more explicit, we define a compact reference architecture that links Layers 1–4 (Table 1). Let $x_{t}$ denote the multimodal sensor observations acquired over time t, including physiological, behavioral, and contextual streams (e.g., HR/HRV, EDA, skin temperature, IMU-derived movement features, task events, and environmental measurements). Let $q_{t}$ denote signal-quality metadata associated with these observations, such as contact confidence, motion artifact likelihood, ambient interference, missingness, and sensor uptime. The purpose of Layer 1 is therefore not only to collect raw data, but to produce paired outputs $\left(x_{t},q_{t}\right)$, where data quality is treated as an explicit model input.

Layer 2 maps these observations into a latent state estimate $z_{t}$, where $z_{t}$ represents the user’s probabilistic physiological or cognitive state (e.g., fatigue, heat strain, stress, cognitive load, or motor instability). Formally, the goal is not a deterministic label, but an estimate of the posterior $p(z_{t}|x_{1:t},q_{1:t},c_{t})$, where $c_{t}$ denotes contextual variables such as task phase, environmental conditions, role requirements, and individualized baseline information. In this framing, uncertainty arises from at least two sources: measurement uncertainty (e.g., noisy or incomplete sensing) and model uncertainty (e.g., distribution shift or limited support from the training data). The Layer 2 output should therefore include: (i) a state estimate, (ii) an uncertainty value or interval, (iii) signal-quality and drift indicators, and (iv) an abstention option when confidence is insufficient for safe recommendation.

Layer 3 receives a structured state representation rather than raw sensor data. Let $s_{t}=\{z_{t},u_{t},d_{t},a_{t},c_{t}\}$, where $u_{t}$ denotes uncertainty, $d_{t}$ denotes a drift or out-of-distribution flag, $a_{t}$ denotes model availability/abstention status, and $c_{t}$ denotes relevant contextual constraints. The role of Layer 3 is to transform $s_{t}$ into a bounded set of candidate interpretations or actions $r_{t}$ using grounded reasoning over trusted local knowledge sources, operating procedures, and task constraints. Importantly, this layer should be schema constrained: outputs should specify the recommended option(s), rationale, confidence/uncertainty statement, and source provenance, rather than unrestricted free-form text.

Layer 4 governs whether, when, and how the output of Layer 3 should be presented to the user. Let $\pi_{t}$ denote an attention-control policy that maps the current system state, user state, task demands, and recommendation urgency onto an intervention decision $y_{t}$. This decision may include whether to alert, defer, query for clarification, log silently, or abstain from action. The central design objective is not simply to maximize detection sensitivity, but to balance expected utility against cognitive cost, interruption burden, and the risk of over-reliance.

Several assumptions underlie this reference architecture. First, sensor observations in field settings are expected to be noisy, incomplete, and context dependent. Second, user baselines and task environments are heterogeneous, necessitating personalization and shift detection. Third, hardware constraints matter: feasible deployment depends on bounded latency, memory, and power budgets, which may require compression, quantization, duty cycling, or fallback to simpler models. Fourth, because deployment may occur in high-stakes settings, the system must preserve uncertainty, support abstention, and remain auditable across all layers. We emphasize that this formulation is not intended as a finalized implementation specification, but as a common scaffold for comparing candidate designs and identifying where technical and human-factor risks enter the pipeline.

## 3. On-Person Cognitive Co-Pilots: A Framework

By cognitive co-pilot, we refer to an on-person, context-aware decision-support system that continuously integrates multimodal sensing, probabilistic state estimation, and grounded reasoning to help a user interpret their current condition, receive contextualized recommendations, and select appropriate actions. Traditional status monitoring dashboards often display signals, summary scores, and threshold alarms. A cognitive co-pilot is designed to reduce interpretive burden by translating uncertain physiological and contextual signals into structured, actionable guidance while preserving human autonomy. As such, a cognitive co-pilot minimally includes: (a) quality-aware sensing, (b) probabilistic state estimation with uncertainty, (c) drift and out-of-distribution detection, (d) grounded reasoning over trusted rules or references, and (e) an explicit intervention policy governing when and how to advise the user and provide recommendations. Optional capabilities include natural language dialog (i.e., a method to prompt and chat), adaptive personalization, user-tunable thresholds, retrieval over historical personal data, and an audit mode that enables after-action explanation.

Such a system does not replace human judgment; rather, it functions as a bounded reasoning partner that manages uncertainty, timing, and attention in support of safe and effective performance. This is realized via a layered loop that couples sensing, inference, reasoning, and presentation with explicit human roles. This framework is depicted in Figure 1.

In addition to Layers 1–4, the framework emphasizes a human-in-the-loop feedback cycle. The user injects context (e.g., task phase, constraints, goals), queries rationale, and provides feedback that can tune thresholds (but within a bounded range), modalities, and (in some cases) local personalization. This is the mechanism by which trust is calibrated and autonomy is preserved.

## 4. Research Agenda and Future Directions

At the sensing layer, the frontier is the deployable validity of sensors. Sweat and tear sensing are advancing quickly, yet field reliability depends on sampling, contamination control, and the interpretability of analytes in context [32,95]. Some sensing modalities, such as heart rate, inertial motion sensing, and skin temperature, are already routinely deployed in ICE environments, including military training, industrial safety, and endurance operations; for these sensors, the research emphasis has shifted from basic capability to ruggedization, long-duration reliability, and integration with quality-aware inference pipelines. Energy autonomy is similarly a gating issue: batteries constrain wear time and user acceptance, motivating hybrid harvesting approaches (triboelectric, piezoelectric, thermoelectric) and energy-aware duty cycling [96,97,98]. A realistic path forward is to treat biochemical sensing as opportunistic and complementary in the near term; they are valuable when available, but not required for baseline operation, while investing in signal-quality detection and artifact modeling across all modalities.

At the signal-to-state layer, the central problem is generalization under shift [45,99,100]. Physiological baselines vary widely, and operational contexts induce systematic deviations (heat, dehydration, altitude, stimulants, sleep debt). Models must therefore be personalized without overfitting, and they must detect when deployment conditions fall outside the training envelope. Federated learning offers one route to improvement without centralizing raw data, but production systems will also need calibration monitoring, adversarial robustness, and audit trails that can withstand scrutiny after adverse events [54].

At the reasoning layer, the research problem is to make the LLM safe and grounded. Retrieval-augmented patterns can constrain generation to vetted sources, but only if retrieval itself is governed: which documents are authoritative, how they are updated, and how conflicts are resolved [72,73,101]. Quantization and compression enable local deployment but may alter failure modes in subtle ways, particularly for uncertainty communication and multi-step reasoning [81]. Methods like generative pre-trained transformer quantization (GPTQ) and activation-aware weight quantization (AWQ) provide practical compression tools, but safety-critical evaluation must move beyond benchmark accuracy to measure hallucination under ambiguous inputs, brittleness under adversarial prompts, and consistency across repeated queries [102,103].

At the presentation layer, the research challenge is when and how to intervene. We already know from alarm fatigue research that frequency and specificity shape compliance, and from automation bias research that authoritative phrasing can degrade verification [82,84]. What remains underexplored is a principled mapping from *recommendation type × task phase × workload × modality constraints* to an intervention policy that preserves performance. This is where simulation-based testing, human-subject experiments, and field trials must converge. The system should be evaluated not only on classification accuracy, but on downstream outcomes: error rates, response latency, workload, trust calibration, and skill retention over time.

### 4.1. Transitioning Research from Lab to Field

**Figure 2:**
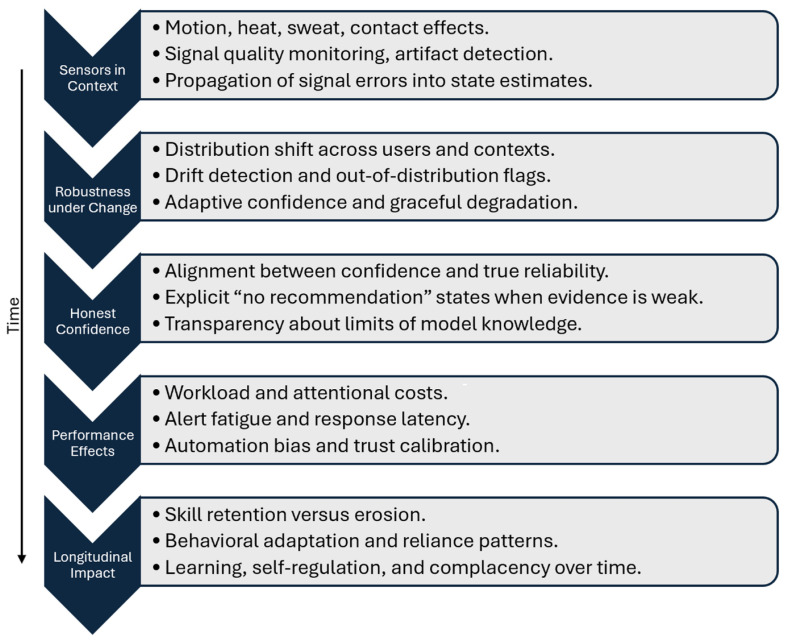
Over time, evaluation should extend from sensor validity in real conditions to robustness and drift awareness, calibrated uncertainty and abstention, human performance effects, and longer-term impacts on skill and reliance.

To support translation beyond this layered framework, cognitive co-pilot systems should be evaluated with a suite of criteria that extends beyond traditional model accuracy or classification performance, as depicted in Figure 2.

First, validation must include sensor performance under realistic operational conditions, explicitly testing robustness to motion, heat, sweat, poor contact, and environmental interference. Laboratory accuracy is insufficient if signal integrity collapses in the field; therefore, performance reporting should include quality-aware metrics, failure characterization, and the downstream consequences of degraded sensing on state estimation and recommendations.

Second, evaluation must examine robustness under changing conditions, including distribution shift, user variability, and evolving operational contexts. Systems should demonstrate not only stable performance across environments, but also the capacity to detect drift, flag out-of-distribution inputs, and appropriately adjust confidence or abstain. This moves evaluation from *does it work?* to *does it know when it might not work?* This is a distinction that is central to trust.

Third, uncertainty calibration should be treated as a first-class outcome. A useful cognitive co-pilot is one that communicates confidence transparently and appropriately limits its own authority. Evaluation should therefore test whether confidence estimates align with true reliability, whether the system appropriately produces “no recommendation” states when evidence is weak, and whether uncertainty information actually improves downstream human decision quality [74,94].

Fourth, translational evaluation must include human outcomes, not just model outputs. This includes workload, response latency, alert fatigue, automation bias, trust calibration, and adherence to recommendations. A system that improves classification accuracy but increases cognitive burden or promotes over-reliance may degrade real-world performance [91]. Accordingly, experimental designs should pair technical validation with human-subject testing that measures how the system changes behavior, attention allocation, and decision processes during representative tasks.

Finally, longer-term effects must be examined. Continuous decision support can reshape user expectations, skill retention, and monitoring behavior over time. Evaluation should therefore extend beyond short trials to assess whether systems promote complacency, erode expertise, or instead support learning and adaptive self-regulation. Longitudinal deployment studies, after-action review analyses, and training-transfer assessments will be essential to determine whether cognitive co-pilots function as sustainable augmentation tools (i.e., not as short-term performance crutches).

As an illustrative validation path, future implementations of this framework could be evaluated in a staged manner. A first phase should quantify sensing and inference performance under controlled manipulations of motion, heat, sweat, poor contact, missingness, and environmental interference, with repeated runs used to characterize variance, calibration, and failure modes. A second phase should evaluate robustness under distribution shift by varying user characteristics, task phases, environmental conditions, and operational tempo, with reporting that includes dispersion, confidence intervals, abstention rates, and drift-detection behavior. A third phase should test downstream human effects in representative scenarios, such as navigation, decision-making, resource allocation, monitoring, triage, or multitask simulations, using outcomes such as decision accuracy, response latency, workload, trust calibration, alert adherence, and evidence of automation bias or over-reliance. Especially for high-stakes use, validation should be designed to determine not only whether the system performs well on average, but whether it remains reliable, honest under uncertainty, and beneficial to human performance across repeated use and heterogeneous conditions. This broader evaluation approach reframes success as demonstrable improvement in human–system performance over time.

### 4.2. Illustrative Edge Deployment Scenario

To make the framework more concrete, consider an illustrative deployment in which a wearable device (e.g., a smartwatch or chest-worn node) performs continuous sensing and lightweight preprocessing, while a paired companion device (e.g., a smartphone, body-worn computer, or vehicle-mounted edge processor) performs state estimation and bounded reasoning. This distinction is important because the proposed framework is not equally implementable on all hardware classes today [77,78]. In near-term deployments, watch-class and sensor-node devices are well suited for continuous acquisition, short-window feature extraction, and signal-quality estimation, but are generally not the preferred location for persistent contextual reasoning with retrieval and language models. By contrast, phones and body-worn computers can more realistically support recurrent probabilistic inference, short-term history storage, retrieval over local reference materials, and event-triggered reasoning.

In such a configuration, Layer 1 would run continuously on the wearable and sample a modest multimodal set such as PPG-derived cardiovascular features, skin temperature, IMU-derived movement features, and task-event metadata, while also generating signal-quality indicators related to contact quality, motion artifact, and missingness. Rather than assuming continuous transmission of raw data, the wearable could compute short-window features and quality summaries locally and transmit those at a lower update rate when needed. This keeps battery and bandwidth demands within realistic limits for persistent use.

Layer 2 would run on the companion device using a compact personalized model that updates a state representation containing variables such as fatigue likelihood, thermal strain likelihood, workload estimate, uncertainty, and drift status. The practical requirement at this layer is not maximal model complexity, but stable recurrent inference with low enough latency to support decisions during ongoing activity. In many plausible deployments, this means sub-second to few-second update cycles, modest memory requirements, and explicit fallback behavior when computation, signal quality, or confidence is limited.

Layer 3 would also likely run on the companion device or another local edge processor, but unlike Layers 1 and 2, it would be invoked intermittently rather than continuously. In practice, this layer is most realistic today when it is bounded: triggered by a threshold crossing, user query, or change in task context; constrained by retrieval over trusted local documents or rules; and required to produce structured outputs rather than unconstrained free text. A compressed local LLM, a smaller reasoning model, or a hybrid rules-plus-retrieval pipeline may all be viable implementations depending on hardware resources and risk tolerance [64,77]. Full, always-on free-form language reasoning on watch-class hardware should not be treated as the default target architecture.

The central architectural implication is that computational burden should be partitioned across layers according to operational role. Continuous sensing and preprocessing should remain extremely lightweight and power efficient. Recurrent state estimation must remain computationally stable enough for persistent use on mobile hardware. Contextual synthesis and explanation should be event-triggered, bounded, and reserved for cases in which they add value beyond simpler decision logic. In this manner, latency, memory, and energy budgets need not be identical across layers; instead, each layer should be designed according to both its temporal demands and the consequences of delay or failure.

From an implementation standpoint, the framework is best interpreted as a tiered architecture with different levels of near-term feasibility. Today, Layer 1 is clearly implementable on watch- and wearable-class devices; Layer 2 is realistically implementable on phones and other companion processors; and Layer 3 is implementable locally only when compressed, retrieval-bounded, and event-triggered. The most resource-intensive functions should therefore migrate to the most capable local device available, while preserving low-latency operation and minimizing off-person data transfer.

We emphasize that this scenario is illustrative rather than definitive. The purpose of this example is not to claim that a single hardware stack is universally sufficient, but to show that end-to-end feasibility depends on careful function partitioning, bounded local operation, and explicit fallback behavior when computing, energy, or confidence are limiting. As summarized in Table 2, the framework partitions tasks based on what is realistically implementable today, their temporal requirements, computational pressure, and appropriate fallback behavior. In practice, the layered framework should be interpreted as a distributed local architecture in which the most time-sensitive and power-constrained functions remain closest to the sensor, while the most memory-intensive and explanation-oriented functions are shifted to the most capable local processor available.

### 4.3. Illustrative End-to-End Walkthroughs

To clarify how the framework operates across layers, we illustrate several end-to-end examples. These examples are not intended as validated deployment claims or optimized policies. Rather, they are intended to show how the proposed architecture transforms sensed signals into probabilistic state estimates, integrates those estimates with local context and trusted guidance, and then applies an intervention policy that may *alert*, *defer*, or *abstain* depending on confidence and operational conditions.

In our first example (alert), consider a user engaged in sustained physical work in a hot environment while wearing a wrist- or chest-based sensing platform and carrying a paired local companion device. Layer 1 acquires multimodal signals including PPG-derived cardiovascular features, skin temperature, IMU-derived movement patterns, and task-event metadata, while also generating signal-quality indicators. Layer 2 integrates these observations with individualized baseline information and current task context to estimate an elevated posterior probability of thermal strain and fatigue, with acceptable signal quality, no drift flag, and sufficiently narrow uncertainty bounds. Layer 3 then retrieves trusted local guidance relevant to heat strain mitigation, task constraints, and available intervention options, and produces a structured recommendation such as: elevated heat strain likelihood relative to baseline, confidence moderate-to-high, recommend short cooling/rest opportunity at next feasible task boundary, reassess within 10 min. Layer 4 evaluates the urgency of the condition against task phase and interruption cost, and delivers a brief cue through a low-burden modality such as haptic feedback with a short visual or auditory explanation. In this example, the system functions not as a dashboard that merely reports physiological values, but as a bounded decision-support partner that links uncertain state estimates to context-sensitive options.

In our second example (defer), consider a user performing a visually demanding or time-sensitive task in which interruption itself may degrade performance, such as air traffic control, piloting, or targeting. Layer 1 detects physiological and behavioral changes consistent with increasing workload or stress, and Layer 2 outputs an elevated stress posterior with moderate confidence, but the inferred task phase indicates that the user is currently in a critical interval with high interruption cost. Layer 3 retrieves relevant operational guidance and produces a structured recommendation indicating that monitoring is warranted but immediate interruption is not preferred unless the state worsens or persists. Layer 4 therefore selects a deferred policy rather than an immediate alert, logging the state change and delaying any overt cue until workload proxies decrease or the task phase changes. In this example, the system uses an explicit attention policy to determine that the correct action is to defer.

In our third example (abstain), we illustrate why abstention is a necessary system behavior rather than a failure mode. Suppose a user is moving vigorously through complex terrain with poor sensor contact, high motion artifact, and physiological patterns that differ substantially from the data on which the model was trained. Layer 1 detects degraded signal quality and missingness. Layer 2 produces a weak or unstable posterior, widened uncertainty, and an out-of-distribution or drift flag. Under these conditions, Layer 3 should not generate a strong recommendation from ambiguous evidence. Instead, the reasoning layer either remains inactive or produces only a structured status such as insufficient confidence for recommendation, signal quality degraded, request sensor check or recalibration, continue monitoring. Layer 4 then withholds an actionable cue and instead logs the event, requests improved sensor fit, or falls back to a simpler monitoring policy. This example is important because a trustworthy cognitive co-pilot must preserve uncertainty and support explicit abstention when evidence is weak, rather than converting low-quality inputs into overconfident advice.

These walkthroughs illustrate three distinct but necessary outcomes of the framework: actionable recommendation, context-sensitive deferral, and explicit abstention. The architectural point is that all three outcomes can be appropriate depending on signal quality, posterior uncertainty, task context, and the cost of interruption. A useful cognitive co-pilot is therefore not defined by how often it generates recommendations, but by whether it generates the right level of intervention (if any) for the available evidence and operational conditions (Table 3).

### 4.4. Practical Limitations and Deployment Constraints

Several practical limitations should be considered prior to deployment. First, scalability may be limited by heterogeneity across users, contexts, and missions. Physiological baselines differ across individuals, and field conditions introduce systematic shifts through heat, dehydration, fatigue, sleep loss, altitude, stimulants, motion artifact, and evolving task demands [104,105]. As a result, a model that appears accurate in one context may degrade substantially in another unless calibration monitoring, shift and drift detection, and abstention are treated as core system functions.

Second, robustness to noise and missingness remains a central constraint. To combat signal degradation in the field, modern systems are moving toward “quality-aware” processing. Technical frameworks now utilize modulation spectral representations to generate signal-quality metadata, allowing the system to adjust enhancement strategies “on-the-fly” based on the detected noise level [106]. Additionally, novel neural network training methods, such as opportunistic teacher-forcing, allow models to maintain reasoning quality even when up to 80% of the physiological data is missing or contaminated [107].

Third, deployment on the edge requires sophisticated resource orchestration. Recent architectures like Synergy enable on-body AI by distributing model workloads across tiny hardware accelerators, optimizing for strict energy and latency budgets [108]. For more complex tasks, “offload shaping” strategies can be used to determine which functions should be processed locally and which should be sent to companion devices to minimize bandwidth and power consumption [109].

Fourth, in safety-critical or industrial environments, purely generative models are often deemed too risky due to non-deterministic outputs. Current state-of-the-art architectures employ a hybrid approach: deterministic rule-based agents enforce hard safety constraints and “interlocks,” while LLMs are reserved for higher-level diagnostic interpretation and natural language interaction with the user [110]. This knowledge-infused design ensures that AI behavior remains predictable and justifiable [111].

Fifth, the attentional action layer can be formalized more rigorously than a simple alerting heuristic. The decision of when to alert a user is increasingly modeled as a formal safety-control game. These frameworks use partially observable stochastic models to balance the utility of an intervention against the “cost” of auditing or human interruption [112]. Integrating generative AI with Instance-Based Learning (IBL) cognitive models further allows systems to better mirror human decision-making processes, enabling more symbiotic human-AI teaming [113].

Finally, the framework requires validation at the level of human–system performance, not only technical component accuracy; indeed, validation is shifting from technical accuracy toward human-centric metrics. The SHAPE-AI tool was recently developed as a standardized tool to monitor how AI affects situational awareness, workload, and trust calibration in real-time clinical settings [114]. Comprehensive meta-analyses suggest that longitudinal validation and participatory design are essential to ensure that explainable AI (XAI) actually improves decision quality without introducing automation bias [115]. In this sense, the most important open question is not whether the system can generate recommendations, but under what conditions it improves human judgment safely and reliably [116].

## 5. Governance, Privacy, and Autonomy

Local processing reduces risk, but it does not eliminate it. Devices can be stolen, compromised, or coerced. Occupational deployments also raise governance questions that are qualitatively different from consumer wellness: who owns the data, who can access derived readiness estimates, and what constitutes meaningful consent when use is mandated [117,118,119]. For example, derived readiness estimates may be interpreted as objective medical facts, used to restrict assignments, or inadvertently incorporated into disciplinary or performance processes.

Two existing frameworks are immediately relevant. The National Institute of Standards and Technology (NIST) AI Risk Management Framework (AI RMF 1.0) emphasizes mapping and measuring risks across the AI lifecycle and aligns trustworthiness with characteristics such as validity, reliability, safety, transparency, accountability, and privacy [120]. The NIST Privacy Framework similarly provides a structure for identifying and managing privacy risk as an enterprise concern. These can inform technical architecture (data minimization, retention limits, access controls, audit logs) and interaction policy (what is shown to whom, when, and why) [121].

From a technical standpoint, privacy-preserving analytics may include secure enclaves and trusted execution environments (TEEs) for data-in-use protection and differential privacy for aggregated reporting [122]. But the ethical core remains sociotechnical: continuous monitoring can easily drift from safety enhancement into surveillance [123]. The only stable boundary is explicit governance: clear purpose limitation, role-based access, transparent user-facing explanations, and avenues for contesting or overriding system-driven inferences [124].

Finally, autonomy is preserved by designing the human-in-the-loop framework so that the human remains an agent in the interaction. That implies opt-out pathways when feasible, graded control over modalities and thresholds, and training that teaches users when the system is competent and when it is not; these are explicit responses to the well-documented risks of complacency and automation bias [125,126,127].

## 6. Conclusions

The convergence of pervasive biosensing, edge computing, and locally hosted LLMs makes a new class of systems plausible: on-person cognitive co-pilots that reduce cognitive burden by translating multimodal physiology into context-aware, actionable guidance. The technical novelty is not in any single sensor or model. Rather, it is in the coupling of probabilistic state estimation, grounded reasoning, and attentional interface design under real operational constraints. Edge deployment is central because it enables low-latency support in connectivity-poor settings and supports privacy-preserving governance by minimizing off-person data flow [19,20].

The research agenda is therefore necessarily multidisciplinary: sensor engineering for field validity, machine learning for calibrated inference under shift, LLM systems engineering for grounded reasoning under compression, and cognitive engineering for interventions that preserve situational awareness and autonomy. If that integration is performed well, cognitive co-pilots can become trusted partners rather than noisy dashboards; they will augment but not replace the human capacity to perform safely and effectively when conditions are at their most challenging.

## Figures and Tables

**Table 1:** The four framework layers, their functional roles, and formal input variables and outputs.

Layer	Functional Role	Input Variables	Output Formalization
Acquisition	Quality-aware data collection	$\left(x_{t},q_{t}\right)$	Paired output where quality is an explicit input
Estimation	Probabilistic state mapping	$x_{1:t},q_{1:t},c_{t}$	$\mathrm{Posterior}\;\mathrm{distribution},\;p(z_{t}|x_{1:t},q_{1:t},c_{t}),\;\mathrm{and}\;\mathrm{uncertainty}\;u_{t}$
Reasoning	Grounded interpretation	$s_{t}=\{z_{t},u_{t},d_{t},a_{t},c_{t}\}$	$r_{t}$: Schema-constrained actions or interpretations
Policy	Attention-control and gatekeeping	$s_{t},r_{t}$, user state, task demands	$y_{t}$: Intervention decision (e.g., alert, defer, abstain)

**Table 2:** Illustrative computational responsibilities, feasibility, and resource considerations across framework layers.

Metric	Layer 1 (Sensing)	Layer 2 (State Estimation)	Layer 3 (Reasoning)	Layer 4 (Intervention Policy)
Primary role	Signal acquisition, preprocessing, signal-quality flags	Probabilistic state estimation, uncertainty, drift detection, personalization within bounds	Contextual synthesis, explanation, option generation, retrieval-grounded recommendation	Decide whether, when, and how to cue and advise user.
Most realistic device location today	Watch, chest-worn node, textile node, or other wearable sensor	Smartphone, body-worn computer, vehicle-mounted edge processor	Smartphone, body-worn computer, or other local edge processor	Likely same device as Layer 2 or 3.
Near-term implementation status	Clearly implementable today	Realistically implementable today on companion hardware	Implementable today only when bounded, compressed, and event-triggered	Implementable today as lightweight gating logic.
Temporal profile	Continuous or high-frequency sampling	Recurrent low-latency updates	Intermittent and event-triggered	Near-immediate once upstream outputs are available.
Typical latency target	Milliseconds to sub-second preprocessing windows	Sub-second to few-second state updates	Second-level acceptable in many use cases if not safety-critical; should be faster when urgency is high	Low-latency cue selection once recommendation state is available.
Main memory/compute pressure	Very limited memory; lightweight DSP/feature extraction only	Compact predictive model, short-term history, calibration/drift checks	Highest memory burden; retrieval store, compressed model, schema enforcement, tool use	Minimal additional compute beyond policy evaluation.
Main power pressure	Highest sensitivity to battery drain and duty cycle	Moderate; repeated on-device inference must remain stable over prolonged use	High per-call energy cost but reduced by intermittent use	Low, provided cueing logic remains simple.
What should usually not run here	Full contextual reasoning, persistent retrieval, large-model inference	Heavy free-form generation if tighter real-time inference is required	Continuous always-on sampling or raw-signal preprocessing	Complex interpretation without uncertainty and drift inputs.
Preferred implementation style	Lightweight signal processing, feature extraction, compression, local quality estimation	Compact probabilistic model, bounded personalization, abstention and drift logic	Compressed local LLM, small reasoning model, rules + retrieval hybrid, strict schemas	Rule-based or utility-based gating policy.
Fallback under constraint	Lower sampling rate, fewer channels, more buffering, reduced transmission	Lower update frequency, simpler model, wider uncertainty, increased abstention	Template-based logic, rule-based heuristics, deferred explanation, abstention	Silence/defer cue, request recalibration, or log only.
Conceptual vs implementable distinction	Largely implementable with current wearables	Largely implementable on current companion devices	Partly implementable now; strongest claims remain conditional on bounded local reasoning and compression	Implementable now if framed as a lightweight attention policy.

**Table 3:** Illustrative end-to-end outcomes across framework layers.

Example Outcome	Layer 1 Inputs	Layer 2 Outputs	Layer 3 Outputs	Layer 4 Action
Alert	PPG, skin temperature, IMU, task context, acceptable quality	Elevated fatigue and thermal strain posterior, moderate-to-high confidence, low drift	Retrieved local heat guidance, structured recommendation to rest and reassess	Brief haptic or audio cue at feasible task boundary
Defer	Physiological arousal and task-demand indicators	Elevated stress and workload posterior, moderate confidence	Monitor and defer recommendation due to task phase	No immediate alert, delay cue until lower-demand period
Abstain	Poor contact, motion artifact, missingness, unusual context	Wide uncertainty, unstable posterior, drift flag	No strong recommendation, request recalibration, sensor check	Abstain, log event, or fall back to simpler monitoring

## Data Availability

Not applicable.

## Abbreviations

The following abbreviations are used in this manuscript:

AI	Artificial Intelligence
AWQ	Activation-Aware Weight Quantization
CNNs	Convolutional Neural Networks
EDA	Electrodermal Activity
EEG	Electroencephalography
EMG	Electromyography
GRUs	Gated Recurrent Units
GPTQ	Generative Pre-trained Transformer Quantization
HR	Heart Rate
HRV	Heart Rate Variability
ICE	Isolated, Confined, and Extreme (environments)
IMU	Inertial Measurement Unit
LLM	Large Language Model
LSTMs	Long Short-Term Memory networks
NIST	National Institute of Standards and Technology
PPG	Photoplethysmography
RAG	Retrieval-Augmented Generation
RMF	Risk Management Framework
SOPs	Standard Operating Procedures
SpO_2_	Peripheral Capillary Oxygen Saturation
TEEs	Trusted Execution Environments
TinyML	Tiny Machine Learning
